# Different Patterns of Relationships Between Principal Leadership and 15-Year-Old Students’ Science Learning: How School Resources, Teacher Quality, and School Socioeconomic Status Make a Difference

**DOI:** 10.3389/fpsyg.2020.02257

**Published:** 2020-08-27

**Authors:** Cheng Yong Tan, Peng Liu, Wai Lun Vincent Wong

**Affiliations:** ^1^Faculty of Education, The University of Hong Kong, Pokfulam, Hong Kong; ^2^Faculty of Education, University of Manitoba, Winnipeg, MB, Canada

**Keywords:** academic achievement, learning enjoyment, PISA, principals, school context, school leadership

## Abstract

The present study critically evaluates whether school leadership influences student learning homogenously regardless of school contexts. It examined relationships between four principal leadership variables (envisioning, instructional management, promoting professional development, empowerment) and two types of student outcomes (enjoyment in learning science, science achievement) in different school contexts [in terms of the availability of science resources, quality of science teachers, and school socioeconomic status (SES)]. The sample comprised 248,620 students and 9,370 principals in 35 developed countries who participated in the Programme for International Student Assessment (PISA) 2015. Latent profile analysis (LPA) showed that schools operated in three types of school contexts with different levels of science resources, proportion of quality science teachers, and school SES. There were also differences in the pattern of leadership practices across the three types of school contexts. Three-level hierarchical linear modeling (HLM) showed that among the four leadership variables, only instructional management was positively associated with students’ enjoyment of science in schools with less science resources and quality science teachers. Therefore, instructional management had compensatory effects for students in less-endowed schools. In contrast, principal leadership related to envisioning, teacher professional development, and empowerment was not positively related to students’ science learning in all three school contexts.

## Introduction

There is a clarion call for school leadership researchers to pay more attention to understanding school contexts and contextualize research in these contexts that school leadership is enacted (Close and Raynor, 2010; Hallinger, 2018). There are many aspects that collectively characterize the complex environments that schools operate in but there are three contextual variables that are especially important because of their proximity to teaching-and-learning. These aspects are namely, adequacy of school resources, quality of teachers, and average school SES level. Indeed, some schools may be more ready to implement leader-initiated changes to improve student learning because teachers are qualified and they embrace student-centered pedagogies (Ingvarson and Rowley, 2017), there are updated resources to support teaching-and-learning (Cohen et al., 2003), and students’ parents have the resources to support school programs (Archer et al., 2015; Tan, 2018b).

The inexorable association between school leadership and contexts has two implications. First, school leaders have to arguably adapt to their contexts. For example, the contingency opportunities theory argues that the agency and effectiveness of leadership depends on environmental opportunities and challenges (Wasserman et al., 2010). Specifically, the theory asserts that leaders adapt organizational variables to their environments and that challenges can impede leadership imperatives. In the context of schools, principals have to adjust their leadership priorities to capitalize on opportunities and address challenges that impact teaching-and-learning. Second, school leadership practices may vary in their effectiveness according to contextual conditions.

Notwithstanding these implications, we do not have an informed understanding of the different types of school contexts that schools are operating in the first place. This may partly explain why there is a paucity of research that examines school leadership in context. To elaborate, a literature review indicates that there are few studies whereby leadership effects are examined for specific school contexts. For example, Jacobson’s (2011) synthesis of findings from the school leadership literature and the International Successful School Principalship Project found that successful principals in challenging, high-poverty schools employed practices such as direction-setting, developing people, and redesigning the organization. They also used distributed leadership and professional self-renewal to sustain their school success over time. Stosich (2016) identified principal leadership and job-embedded support from experts as crucial for teachers undergoing attending professional development to translate their learning to instruction and collaboration in two high-poverty schools in the United States. Day et al. (2009) study of improving schools in England found that heads of schools operating in challenging contexts developed and sustained school policies for pupil behavior, motivation, and engagement; instructional standards; physical environments; teaching-and-learning improvements; and cultures of care and achievement. They also worked closely with parents and the community to improve student outcomes. Notman and Henry’s (2011) qualitative study of six New Zealand primary and secondary school principals showed that principals highlighted the importance of developing learning and social assistance programs to address student and family needs for schools in lower-SES areas. The principals also demonstrated contingent leadership in response to external influences related to community support, inadequacy of financial resources from the central government, and the school’s SES context, in order to sustain their leadership success over time. Walker and Ko’s (2011) study reported that principals in Hong Kong schools leveraged the professional development of teachers and key staff to foster within-school alignment and the congruence between the school and government policies when faced with increasing accountability pressures. Researchers in these studies focus on specific school contexts instead of examining a range of contexts.

There are even fewer studies that compare leadership effects in different school contexts. For example, Hallinger and Murphy’s (1986) study of elementary schools in California found that principals of effective, low-SES schools focused on students’ mastery of basic skills in their school mission, exercised tight instructional control and task orientation, and buffered between home and school to minimize parental involvement. In contrast, principals of effective, high-SES schools focused on students’ academic achievement, exercised low-to-moderate instructional control and relationship orientation, and promoted home-school cooperation. Tan (2018a) compared principal leadership effects on 15-year-old students’ mathematics achievement using international data from PISA 2012. Results showed that the student sample could be divided into three sub-groups (disadvantaged, average, privileged) varying in their SES levels, teacher quality and educational resources in school, and parental academic expectations. Furthermore, principal leadership explained a greater proportion of the between-school achievement variance for disadvantaged as compared to other two sub-groups of students. Specifically, instructional management had the strongest positive association with student achievement for disadvantaged students. The other leadership variables were mostly negatively related to student achievement but the strength of association varied among the different sub-groups of students. Bottery et al. (2008) qualitative study of English headteachers and Hong Kong principals found the leadership of both groups of school leaders was influenced by contextual challenges arising from legislation, government inspection procedures, marketization, parental choice, and competition. For example, English headteachers were more adversely impacted by legislation and tough school inspections than were Hong Kong principals.

The present study addresses these knowledge gaps by identifying a typology of school contexts (as measured by the combination of variables measuring teacher quality, adequacy of school resources, and average school SES level) and examining how relationships between principal leadership and student science learning differ in these different contexts.

## Literature Review

### School Contexts

The literature review first discusses the three school context variables and their relationships with teaching and learning. Context is an elucidative element of school effectiveness and improvement at levels of both the school and entire education system (Harris et al., 2015). According to Hallinger et al. (1996), school context can be viewed as having three main aspects: school resources, teacher quality, and student SES.

The first aspect of school context is school resources. Findings from earlier studies showed that there is a consistently positive relationship between school resources and student achievement (Greenwald et al., 1996; Houtenville and Conway, 2008). According to Reynolds (2010), school effectiveness and improvement research has linked promoting school-community collaboration and developing the school culture (both contributing indirectly to school resources) to school turnaround and improvement. As for teacher expenditures (another investment in school resources), scholars including Hedges et al. (1994); Greenwald et al. (1996) believed that there is a strong and significant relationship between such expenditures and student achievement. However, there have also been differing voices in this discussion. Some researchers argued that the effect of school resources can be negligible or even negative (Hanushek, 1996; Häkkinen et al., 2003). After analyzing data from a large sample of matriculation examination scores of Finnish senior secondary school students from 1990 to1998, Häkkinen et al. (2003) concluded that changes in teaching expenditure did not make any difference to students’ test scores. In fact, evidence did not support the idea that low levels of student performance in poor school districts was related to inadequate spending levels. Students in rural areas might be associated with higher per capita teaching expenditure but their performance still lagged that of students from urban areas.

Teachers’ teaching experience and teaching quality, the second aspect of school contexts according to Hallinger et al. (1996), also has an influence on students’ learning outcomes. A meta-analysis by Davis-Beggs (2013), synthesizing results from studies published from 1996 to 2009, reported a significant positive relationship between teachers’ teaching experience and student achievement. Moreover, she suggested that the quality of professional development and the coherence of programs are the strongest predictors of high school students’ academic achievement.

Student SES, Hallinger et al.’ (1996) third aspect of school contexts, has also been regarded as the one of the main factors contributing to student achievement (Organization for Economic Co-operation and Development [OECD], 2004; Perry and McConney, 2010). Many reasons explain this relationship, such as demographics and other characteristics of students’ parents, school community, and peers. For example, Zhang et al. (2011) showed that high-SES students had higher parental engagement than low-SES students, and that the level of parent engagement made a difference to student learning. As for school SES, Van Ewijk and Sleegers (2010) suggested that schools with high SES have higher average student achievement scores, so grouping high-SES students together could result in better student achievement than would be expected from the individual students alone.

The literature examined above has shown that school contexts, including factors such as school resources, teachers’ quality and teaching experience, and students’ SES, are crucial to student learning. Therefore, it is important for researchers to identify and account for school contexts in their studies.

### Principal Leadership

The principal plays an important role in improving the quality of education in a school (Gurr et al., 2006; Waldron and McLeskey, 2010). Principal leadership can influence the trajectory of a school’s development. The literature has emphasized the impact that school principals have on their students’ learning and achievement through their leadership behaviors (Murillo and Hernández-Castilla, 2015; Day et al., 2016). Studies suggest that principal leadership is characterized by two major foci, namely instructional management and teacher capacity-building (Hoppey and McLeskey, 2013).

Leithwood et al.’ (2006) conceptualization of four principal leadership functions encapsulate these two foci. First, principals galvanize schools’ resources to realize the school vision, mission, and goals. The shared vision and mission enable resources to be aligned to achieve school goals (Murphy and Torre, 2015). For example, principals may focus on developing teachers and designing teachers’ work to achieve school goals. Second, principals manage the instructional program by leading teachers in teaching–and-learning, promulgating effective instructional practices, and emphasizing students’ holistic development (Hitt and Tucker, 2016). Envisioning and instructional management are arguably related to the notion of instructional leadership.

Third, principals facilitate teachers’ professional development. This leadership practice builds on individual teacher strengths and needs, inculcates teacher responsibility for professional development, and cultivates teacher professional learning communities (Opfer and Pedder, 2011; Murphy, 2015). For example, principals can have regular discussions with teachers on instructional effectiveness and challenges faced during teaching. They can also provide professional development support to help struggling teachers (Yariv and Kass, 2019). Lastly, principals empower teachers by promoting collaborative decision-making processes. For example, they can distribute leadership roles to involve teachers in reviewing management practices and contributing to school improvement. Promoting professional development and teacher empowerment are related to the notion of enhancing teacher capacity.

### Students’ Enjoyment of Learning and Academic Achievement

Student learning comprises attitudinal and achievement indicators. Accordingly, the present study focuses on students’ enjoyment in learning science and their science achievement. According to Fredrickson (2001), students who enjoy learning have “the urge to play, push the limits, and be creative” (p. 220). It is reasonable to expect then that students who enjoy their learning in a specific subject may be more interested to learn about different topics in that subject. Students’ enjoyment in science learning is an important variable to study because it predicts their interest in science (Ainley and Hidi, 2014), engagement in science learning (Hampden-Thompson and Bennett, 2013), participation in science extracurricular (Lin et al., 2012), science achievement and career aspirations (Jeffries et al., 2020), and collaborative problem-solving (Camacho-Morles et al., 2019). Additionally, students who enjoy their learning may internalize scientific principles (i.e., epistemological beliefs) more easily and therefore, are expected to have higher levels of science achievement (Acosta and Hsu, 2014).

According to the control-value theory, students’ enjoyment of learning is dependent on their control and value appraisals (Pekrun, 2006; Pekrun et al., 2007). Control appraisals relate to students’ competence perceptions of the degree to which learning outcomes are controllable (e.g., ability self-concepts). Value appraisals comprise intrinsic (e.g., perceiving learning activities as interesting and important in itself) and extrinsic (e.g., perceiving activities as important for achievement or relevant to daily life) components. More generally, students’ achievement emotions can be understood in terms of object focus (activity or outcome), valence (positive or negative), activation (activating or deactivating), and prospective-retrospective dimensions. For instance, students’ enjoyment of science learning comprises positive emotions of pleasure activated through participation in science learning activities. It can be associated with positive anticipation of expecting high test performance in science or retrospective joy after experiencing academic success in science.

### Influence of Principal Leadership on Student Learning

The review will next discuss how principal leadership influences student learning in different contexts. When teachers participate in envisioning led by the principal, they are imbued with a shared sense of purpose, higher academic expectations, and commitment. These attributes, in turn, eventuates in a positive student learning climate in the school (Hendriks and Scheerens, 2013). In a related vein, when principals focus on instructional management, students have more learning opportunities because teachers may employ student-centered pedagogies that promote enjoyment in learning (Hendriks and Scheerens, 2013). Students can further benefit from these leadership practices (envisioning and instructional management) if the school context is favorable (e.g., well-resourced schools with qualified teachers) because they can learn from qualified, motivated teachers who employ engaging instructional practices with requisite, up-to-date educational resources. Students are more likely to receive reinforcement from their parents if the latter are more educated (e.g., higher-SES parents) because the latter are more likely to appreciate the importance of science learning and careers in science, technology, engineering, and mathematics or STEM (Archer et al., 2015).

From the perspective of control-value theory, these principal leadership functions (envisioning and instructional management) and supportive school conditions (well-resourced schools, qualified teachers, and higher-SES parents) are likely to eventuate in higher levels of student-perceived control (e.g., self-regulation and efficacy beliefs; Adams and Olsen, 2017; Zheng et al., 2017) and value (e.g., arising from autonomy support that teachers provide to support student learning; Adams and Olsen, 2019). These enhanced appraisals then enhance students’ enjoyment of learning and achievement in science. Therefore, students whose principals exercise leadership in envisioning and instructional management in favorable school contexts are expected to have more positive science learning attitudes which eventuate in higher levels of science achievement.

Next, teachers in schools whereby principals invest in providing professional development may be more cohesive, professional, competent, and efficacious (Hendriks and Scheerens, 2013). A professional learning community comprising these teachers contributes to the school academic and improvement capacity, thereby benefiting student learning. However, if principals are promoting teacher professional development to address the problem of poor teacher quality (an unfavorable school contextual indicator), it is difficult to predict how this leadership practice will impact student learning for two reasons. First, multiple factors need to accompany teachers’ professional development to contribute to student learning (Opfer and Pedder, 2011). These factors include simultaneous, mutually reinforcing changes in teachers’ professional beliefs and practices after professional development and acquisition of subject-specific pedagogical skills. Therefore, professional development is more likely to be successful if the teacher participants are certified specialist teachers (e.g., qualified science teachers) in the first place. Second, professional development impacts teaching-and-learning positively if there is accompanying organizational support. In science education, if there is a severe deficit in science teacher quality and principals’ science education knowledge, then professional development may not benefit students’ science learning. The latter is evident in Lochmiller’s (2016) qualitative study which demonstrated how high school principals who had limited understanding in mathematics and science education resorted to focusing on pedagogy instead of content when they gave feedback to teachers, relying on their past experience as teachers to inform the feedback provided, and using student assessment to make their feedback more meaningful.

Lastly, teacher empowerment creates positive school conditions (trust, care, risk-taking, continuous learning) that promote student learning (Hunzicker, 2012). Separately, students learn better when they are taught by qualified teachers, a school context variable examined in the present study. To illustrate, Woolnough’s (1994) study of A-level students found that the quality of science teaching was one of the most important variables predicting whether students chose science as a subject. It can be argued that quality science teaching is more likely to come from well-qualified graduate science teachers who have the requisite science expertise and subject affiliation, enthusiasm in their science teaching, ability to contextualize science lessons in daily life, capacity to conduct structured yet stimulating science lessons, and willingness to spend time beyond class to have conversations with students about science. Therefore, we can expect qualified teachers, when empowered by principals, to make better decisions that cater to the learning needs of students. Empowered qualified teachers are also more adept at supporting different aspects of peers’ professional growth such as sharing quality, relevant professional learning and providing support on pedagogical content knowledge issues (Wenner and Campbell, 2017), thereby benefiting student learning. In sum, empowering qualified teachers is expected to benefit students in their learning.

### The Present Study

The present study (a) identifies a typology of school contexts (as measured by the combination of variables measuring adequacy of school resources, teacher quality, and school SES); and (b) examines relationships between four core leadership practices (envisioning, instructional management, promoting professional development, empowerment; Leithwood et al., 2006) and student science learning using data from PISA 2015.

The PISA student sample comprises fifteen-year-old students in participating countries. This sample is appropriate for the present study because most students reach the end of their compulsory education by this age in many education systems (Organization for Economic Co-operation and Development [OECD], 2009), so it is important to ascertain the influence of school variables such as principal leadership and school contexts on the science achievement of this group of students. Students of this age group will also have developed the cognitive capacities to conduct appraisals of competence and value (Pekrun and Stephens, 2012; Pekrun, 2017) and therefore able to report their emotions (e.g., enjoyment of science learning) more accurately (Raccanello et al., 2018).

Principal, as opposed to teacher, leadership is examined as principal leadership constitutes the most important source of leadership in schools. For example, Leithwood and Jantzi’s (2000) study found that principal, instead of teacher, leadership contributed to student engagement in Canadian schools. Day et al. (2009) reported that headteachers are regarded by teachers, governors, and parents as the key source of leadership impacting teaching processes in improving English schools. Principal leadership is measured using Leithwood et al. (2006) four core leadership functions because there is evidence that they characterize leadership practices of most school leaders, including successful principals leading schools in challenging contexts (Day et al., 2009; Jacobson, 2011).

The present study analyzed PISA 2015 which focused on students’ science learning for three reasons. First, understanding how principal leadership and school contexts affect students’ science learning is important given that the need for students to have the requisite literacy in STEM in modern society (Xie et al., 2015). Specifically, it is important to increase students’ science participation to nurture what Irwin (2001) referred to as “science citizens” who can use scientific knowhow and technology to solve daily problems (Claussen and Osborne, 2013). Second, such students are also better placed to exploit opportunities in higher education and occupational markets in STEM disciplines characterizing KBEs in the longer term. Occupational opportunities in STEM are diverse, so students can select specific jobs that match their interests, afford them a better quality of life, and enhance their social mobility (Xie et al., 2015). Third, compared to other subjects such as reading, science learning is more susceptible to school teaching and resources (Reynolds et al., 2014) than family socialization, thereby enabling the present study to unravel contextualized principal leadership effects (if any) on student learning.

Students’ enjoyment in science learning is examined in addition to their academic achievement in line with a more holistic conception of education. Students’ learning attitudes are important because individuals need to be life-long learners in STEM economies and continuous learning requires students to enjoy learning. Furthermore, students’ positive learning attitudes contribute to their academic achievement (Lam and Lau, 2014). Notwithstanding the salience of holistic learning outcomes, there are few studies examining how principals contribute to students’ learning attitudes [e.g., self-regulated learning (Adams and Olsen, 2017), language self-efficacy (Zheng et al., 2017), engagement (Leithwood and Jantzi, 2000), and perceived autonomy support (Adams and Olsen, 2019)]. Therefore, the present study ascertains if principal leadership practices can improve students’ learning attitudes and achievement in science.

Latent profile analysis (Oberski, 2016) is used to empirically derive a typology of schools operating in different contexts in the present study. It achieves this by examining the pattern of contextual variables in the sample, uncovering underlying heterogeneity, and identifying distinct sub-groups of schools varying in the three school contextual variables. This approach is useful because of its objective, data-driven approach and because it allows researchers to simultaneously incorporate multiple, correlated contextual indicators in identifying a typology of schools. The less-effective alternative approach will be to assign schools in the sample to arbitrary sub-groups based on *a priori* considerations one indicator at a time (classifying schools as low-, average-, and high-SES schools or as schools with low, average, or high proportions of qualified teachers) and drawing separate conclusions for each typology of schools.

## Materials and Methods

### Participants

The sample comprised 248,620 students and 9,370 school principals in 35 OECD countries who participated in PISA 2015 (Organization for Economic Co-operation and Development [OECD], 2017). The majority of these students were in Grade 10 (55.9%) with the rest were from Grades 7−13. 69.7% of the schools were public schools while 14.8% were private schools (15.5% of the schools were unclassified). Participating students were selected to represent the complete population of 15-year-old students who were attending public or private schools in grade 7 or higher in the participating countries. PISA 2015 measured 15-year-old students’ proficiency in applying their knowledge and skills learned in science (the focal domain) in addition to reading and mathematics. In addition, PISA 2015 collected background data from students, parents, principals, and teachers, on student/home/family, classroom, and school variables.

### Measures

The present study analyzed data from principals’ and students’ responses to the School and Student Questionnaires, respectively^1^, and students’ science performance^2^. Data on the following PISA 2015 variables were used in the analysis.

#### Science Resource Availability

The availability of science resources in schools (SciRes) was measured by summing up principals’ responses (*Yes*, *No*) to eight items on the availability of resources for the science department (e.g., “Compared to similar schools, we have a well-equipped laboratory.”) The items pertained to equipment in the science department, allocation of extra funding to science teaching, educational levels of science teachers, materials for laboratory and hands-on learning, laboratory support staff, and up-to-date science equipment.

#### Science Teacher Quality

The quality of science teachers (TrQua) was measured by the proportion of science teachers with a bachelor/master and science major qualifications in schools (principal-reported).

#### Principal Leadership

Principal leadership was measured with four scales using principals’ responses to 13 items asking about the frequency of specific leadership behaviors with a six-point scale (1 = *Did not occur*, 2 = *1*–*2 times during the year*, 3 = *3*–*4 times during the year*, 4 = *Monthly*, 5 = *Weekly*, 6 = *More than once a week*). The scales corresponded to the four core principal leadership functions identified by Leithwood et al. (2006). The first scale (Envisioning) measured envisioning (α = 0.99) − principals’ framing and communication of school goals and curricular development − with four items pertaining to principals using student results to develop school academic goals, aligning teachers’ professional development and work to school goals, and discussing school goals with teachers (e.g., “I use student performance results to develop the school’s educational goals.”) The second scale (Instructional-Mgmt; α = 0.98) measured instructional management using three items related to principals promoting research-based teaching practices, praising teachers whose students were learning actively, and emphasizing to teachers the development of critical and social capacities in students (e.g., “I promote teaching practices based on recent educational research.”). The third scale (Professional-Devt; α = 0.98) measured principals’ promotion of teachers’ professional development using three items pertaining to principals taking the initiative to discuss problems teachers encountered in classrooms, paying attention to students’ disruptive behavior, and solving classroom problems with teachers collaboratively (e.g., “When a teacher has problems in his/her classroom, I take the initiative to discuss matters.”). The fourth scale (Empowerment; α = 0.98) measured empowerment − principals’ facilitation of teachers’ participation in leadership − using three items related to principals engaging staff to participate in school decision-making, building a school culture of continuous improvement, and reviewing management practices (e.g., “I provide staff with opportunities to participate in school decision-making.”). These four leadership scales corresponded to the four core principal leadership functions identified by Leithwood et al. (2006). Confirmatory factor analysis (CFA) showed that the four scales explained the variation in the 13 items satisfactorily [χ^2^(59) = 3,627.96, *p* < 0.01; CFI = 0.93; TLI = 0.91; RMSEA = 0.085; SRMR = 0.05].

#### Student and School SES

Student SES (StuSES) was measured by the index of economic, social, and cultural status computed by PISA 2015 (Organization for Economic Co-operation and Development [OECD], 2017). The index represented the first principal component derived from student data on parents’ highest education level, parents’ highest occupational status, and students’ home possessions. Data on parents’ highest education level were derived from student responses on their parents’ highest levels of schooling completed (two questions for each parent). The response categories corresponded to “*no education*,” “*primary education*,” “*lower secondary*,” “*vocational/pre-vocational upper secondary*,” “*general upper secondary and/or non-tertiary post-secondary*,” “*vocational tertiary*,” and “*theoretically oriented tertiary and postgraduate*.” Data on parents’ highest occupational status were derived from students’ responses on the nature of their parents’ main jobs (two questions for each parent). PISA 2015 coded these data and mapped the codes onto the international socioeconomic index of occupational status (Ganzeboom and Treiman, 2003). Data on student home possessions were derived from student responses to three questions asking about the availability of different home resources such as study desk, own room, quiet place to study, computer for study, educational software, Internet connectivity, classic literature, poetry books, art works, books to support study, reference books, dictionary, books on art/music/design, televisions, cars, rooms with bath/shower, cell phones with Internet access, tablet computers, e-book readers, and musical instruments. The present study averaged students’ SES levels within a school to obtain a measure of school SES (SchSES).

#### Student Gender

A variable identifying student gender (Male) was coded 1 and 0 for boys and girls, respectively.

#### Confucian Heritage Culture (CHC)

Countries were coded 1 if they were CHCs (Japan and Korea) and 0 otherwise.

#### Students’ Enjoyment in Learning Science

Dependent variables comprised students’ enjoyment in learning science and their science achievement. Students’ enjoyment in learning science (Enjoy; α = 0.99) was computed from student responses to five items measuring the extent to which they enjoyed learning science using a four-point scale (*Strongly disagree*, *Disagree*, *Agree*, *Strongly agree*). These items pertained to students learning, reading on, and working on science topics (e.g., “I generally have fun when I am learning < broad science > topics.”). CFA showed satisfactory model fit with the five items specified to load on a single latent construct [χ^2^(5) = 6,679.53, *p* < 0.01; CFI = 0.99; TLI = 0.99; RMSEA = 0.076; SRMR = 0.01].

#### Students’ Science Achievement

Students’ science achievement was the focal dependent variable measured in PISA 2015. Students were not administered the complete set of test items by design, and therefore each item had missing responses. This made it impossible to estimate achievement scores for each student. To overcome this limitation, PISA 2015 aggregated the results of individual students to produce scores for groups of students. It also used a set of ten “plausible values” (PV1-PV10) for each student to represent the estimated distribution of science scores of students similar to him or her in terms of responses to the assessment and background items.

### Procedure

PISA 2015 used a two-stage stratified sampling design, with schools first selected from a national sampling frame of schools with probabilities proportional to size and students next selected from within each of the schools (Organization for Economic Co-operation and Development [OECD], 2017). PISA 2015 was sponsored internationally by the OECD. All participating countries followed standardized procedures outlined in the technical standards and manuals provided.

According to Organization for Economic Co-operation and Development [OECD] (2017), PISA was managed by a large international team comprising the PISA Governing Board (PGB), experts in working groups, National project Managers (NPMs). OECD Secretariat, Educational Testing Service (ETS) in the United States, and other external contractors. Specifically, the implementation of PISA was informed by a framework established by the PGB that comprised senior policymakers from all participating countries. The PGS oversaw the establishment of policy priorities and standards for developing indicators, for establishing assessment instruments, and for reporting results. Experts from participating countries formed working groups to relate PISA policy objectives to the best international available technical expertise to ensure that the instruments were internationally valid, culturally sensitive, sound in measurement potential, authentic, and educationally valid. An NPM was appointed in each of the participating countries to ensure that internationally established technical and administrative procedures were adopted. The NPMs developed and validated the assessment instruments and evaluated the survey results, analyses, and reports. The OECD Secretariat was overall responsible for the management of PISA. It provided day-to-day monitoring, provided secretariat services for the PGB, facilitated consensus-building among participating countries, and mediated between the PGB and international contractors. The ETS was responsible for the overall management of external contractors. These contractors were responsible for designing and implementing the surveys.

### Missing Value Imputation

Missing values may compromise estimation efficiency and produce biased results (Cheema, 2014). Therefore, imputation by fully conditional specification was used to address the methodological challenge arising from missing values in the variables in SPSS 25. Imputation of variables with missing values using all other variables as predictors continued until the maximum number of iterations was reached. The imputed dataset then comprised imputed values at the maximum iteration. A set of missing values was imputed separately for school-level variables (SciRes 19.31% missing; TrQua 18.20% missing; Envisioning 12.28% missing; Instructional-Mgmt 12.27% missing; Professional-Devt 12.41% missing; and Empowerment 12.48% missing) and student-level variables (StuSES 2.31% missing; and Enjoy 9.42% missing). There were no missing values for other variables (Male, SchSES, CHC, and SciPV1-SciPV10).

### LPA

Latent profile analysis (Oberski, 2016), using MPlus8, was employed to uncover underlying heterogeneity in schools within the sample and identify distinct groups of schools varying in the three school contextual variables (availability of science resources, quality of science teachers, school SES). There is no clear-cut way of determining the “correct” number of latent classes underlying the population but most analysts make their decision by examining different indicators such as information criteria indicators (Akaike Information Criteria or AIC, Baysesian Information Criteria or BIC, sample-adjusted BIC), entropy, and model parsimony (Nylund et al., 2007). A single-level LPA was performed for the school-level contextual variables because simulation studies have indicated that it yields results similar to those from multilevel LPA as long as the sample size is reasonably large and latent classes are distinct from each other (Park and Yu, 2018). In the context of the LPA for the present study (results to be reported in greater detail later), the total number of schools (9,370) was deemed to be sufficiently large with each latent class having between 1,782 (19.02% of total number of schools) and 5,713 schools (60.97% of schools) and mean levels of the three school context variables varying substantially among the latent classes identified.

### Three-Level HLM

For each latent class of schools, three-level fixed effect HLM with full maximum likelihood estimation and robust standard errors was performed using HLM7.03 to examine relationships between school leadership and student outcomes (Raudenbush and Bryk, 2002). The use of robust standard errors enables unbiased standard errors to be computed even when model assumptions are violated and therefore, mitigates the problem of model misspecification. The four school leadership variables were entered into the models separately as the CFA results indicated high intercorrelations among them (0.58 to 0.90). All independent variables were rescaled by subtracting the grand mean of the entire sample from the respective raw scores for ease of interpretation. After the rescaling, each HLM parameter represents the “effect” of the respective variable for a student with values equal to the grand mean for the other variables. Senate weights for student- and school-level variables were included in the HLM.

Students and schools in each of latent classes were analyzed separately. The following set of nested HLM models was fitted for each student outcome (PV1-PV10, Enjoy). Three sets of nested models were estimated:

• Model 1 − baseline with no predictors;
• Model 2 − random intercepts model with student-level (Male, StuSES) and country-level (CHC) control variables; and
• Model 3 – random intercepts model with Model 2 variables and one of the four school leadership variables (Envisioning, Instructional-Mgmt, Professional-Devt, Empowerment).
• The mathematical formulation for Model 3 predicting students’ science achievement is:

PV_ijk_ = γ_000_ + γ_001_^∗^CHC_k_ + γ_010_^∗^LeadershipVariable_jk_ + γ_100_^∗^MALE_ijk_ + γ_200_^∗^StuSES_ijk_ + r_0*jk*_ + u_00*k*_ + e_ijk_

The mathematical formulation for Model 3 predicting students’ enjoyment of science is:

Enjoy_ijk_ = γ_000_ + γ_001_^∗^CHC_k_ + γ_010_^∗^LeadershipVariable_jk_ + γ_100_^∗^MALE_ijk_ + γ_200_^∗^StuSES_ijk_ + r_0*jk*_ + u_00*k*_ + e_ijk_

for student i from school j in country k. e_ijk_, r_0*jk*_, and u_00*k*_ = level-1,level-2, and level-3 residuals respectively. LeadershipVariable = Envisioning, Instructional-Mgmt, Professional-Devt, or Empowerment.

For the HLM involving students’ science achievement, HLM7.03 estimates parameters for each plausible value separately and averages the ten estimates. It also combines the average of the sampling error from the ten estimates with the variance between the ten estimates multiplied by a factor related to the number of plausible values to yield the measurement error.

## Results

### Typology of Schools

Various information criteria (AIC, BIC, and sample size-adjusted BIC) showed decreasing values when the number of latent classes was increased from 1 to 5 (Table 1). However, the percentage decrease in the information criteria was marginal when the number of latent classes was increased from 3 to 4 (1.04%, 1.00%, 1.02% for AIC, BIC, sample size-adjusted BIC, respectively) as compared to the case when the number of latent classes was increased from 1 to 2 (6.83%, 6.79%, 6.81%, respectively) or from 2 to 3 (5.01%, 4.97%, 4.99%, respectively). Entropy was the highest at.95 for 3 as compared to 1, 2, 4, or 5 latent classes. Therefore, a 3-class solution best characterized the typology of school contexts based on the three contextual indicators. Results of the final class counts and proportions for the latent classes based on their most likely latent class membership showed that 5,713 schools belonged to Class 1 (60.97%), 1,782 schools belonged to Class 2 (19.02%), and 1,875 schools belonged to Class 3 (20.01%). The classification quality was satisfactory as evident by the high mean “dominant” probability (i.e., highest probability of belonging to a class) of 0.98 and 97.6% of the sample having a high dominant probability of at least 0.70.

**TABLE 1:** LPA model fit indicators.

No. of latent classes (n)	Information criteria			Entropy	Percentage decrease in information criteria for n classes as compared to (n-1) classes		
			
	AIC	BIC	Sample size- adjusted BIC		AIC	BIC	Sample size-adjusted BIC
(1)	82,645.75	82,688.63	82,669.56	-			
(2)	76,999.03	77,070.48	77,038.70	0.92	6.83	6.79	6.81
(3)	73,137.87	73,237.91	73,193.42	0.95	5.01	4.97	4.99
(4)	72,374.58	72,503.20	72,446.00	0.92	1.04	1.00	1.02
(5)	71,953.09	72,110.28	72,040.37	0.89	0.58	0.54	0.56

Mean levels for the three school context indicators (Table 2) were all significantly different from zero except for school SES in Class 3 (*p* = 0.69). Schools in Class 1 (named EquippedSch-AveSES) had the highest mean levels of science resources and quality of science teachers and average level of mean school SES. Schools in Class 2 (named NeedySch-LowSES) had the lowest mean levels of science resources, quality of science teachers, and school SES. Schools in Class 3 (named AveSch-HighSES) had average mean levels of science resources and quality of science teachers but the highest mean level of school SES.

**TABLE 2:** Descriptives for latent classes.

	*M* (*SE*) for latent classes		
	Schools with highest levels of resources and most qualified teachers but average SES levels (EquippedSch-AveSES)	Schools with lowest levels of resources, least qualified teachers, and lowest SES levels (NeedySch-LowSES)	Schools with average levels of resources and moderately qualified teachers but highest SES levels (AveSch-HighSES)
Availability of science resources	0.62**(0.00)	0.47**(0.01)	0.56**(0.01)
Quality of science teachers	4.83**(0.01)	0.39**(0.02)	2.65**(0.03)
School SES	−0.06* (0.03)	−0.49**(0.05)	0.02 (0.04)

*p < 0.05, **p < 0.01.

### Comparison of Principal Leadership Among Latent Classes

ANOVA showed that there were overall differences in mean levels of the four school leadership variables among the three latent classes (F for Envisioning = 40.09, *p* < 0.01; F for Instructional-Mgmt = 31.05, *p* < 0.01; Professional-Devt = 3.07, *p* < 0.05; Empowerment = 20.28, *p* < 0.01; Table 3). Tamhane *post hoc* tests indicated that compared to schools in the other two latent classes, schools with the highest levels of resources and most qualified teachers but average SES levels (EquippedSch-AveSES) had the highest levels in all four school leadership variables except for Professional-Devt where there were no differences between EquippedSch-AveSES and schools with lowest levels of resources, least qualified teachers, and lowest SES levels (NeedySch-LowSES) (Mean difference or MD = 0.01, *p* = 0.99). In addition to similar mean levels of Professional-Devt between NeedySch-LowSES and EquippedSch-AveSES, there were no significant differences in mean levels of the four leadership variables between NeedySch-LowSES and schools with average levels of resources and moderately qualified teachers but highest SES levels (AveSch-HighSES) (MD for Envisioning = −0.01, *p* = 0.96; MD for Instructional-Mgmt = 0.01, *p* = 1.00; MD for Profdev = 0.06, *p* = 0.21; MD for Empowerment = 0.02, *p* = 0.96).

**TABLE 3:** Principal leadership for different latent classes.

	Envisioning	Instructional-Mgmt	Professional-Devt	Empowerment
	
	M (*SE*)			
Schools with highest levels of resources and most qualified teachers but average SES levels (EquippedSch-AveSES)	3.47 (0.01)	3.85 (0.01)	4.47 (0.01)	3.95 (0.01)
Schools with lowest levels of resources, least qualifed teachers, and lowest SES levels (NeedySch-LowSES)	3.29 (0.02)	3.67 (0.03)	4.46 (0.02)	3.82 (0.02)
Schools with average levels of resources and moderately qualified teachers but highest SES levels (AveSch-HighSES)	3.30 (0.02)	3.66 (0.03)	4.40 (0.02)	3.81 (0.02)

	**Comparison of mean levels**			

ANOVA (F statistics)	40.09**	31.05**	3.07*	20.28**

	**Tamhane *post hoc* tests (Mean differences)**			

EquippedSch-AveSES vs. NeedySch-LowSES	0.18**(0.02)	0.18**(0.03)	0.01 (0.03)	0.13**(0.03)
EquippedSch-AveSES vs. AveSch-HighSES	0.16**(0.02)	0.19**(0.03)	0.07*(0.03)	0.14**(0.03)
NeedySch-LowSES vs. AveSch-HighSES	−0.01 (0.03)	0.01 (0.04)	0.06 (0.03)	0.02 (0.03)

*^∗^p < 0.05 and ^∗∗^p < 0.01.*

### Comparison of Student Learning Among Latent Classes

Schools with highest levels of resources and most qualified teachers but average SES levels (EquippedSch-AveSES), schools with lowest levels of resources, least qualified teachers, and lowest SES levels (NeedySch-LowSES), and schools with average levels of resources and moderately qualified teachers but highest SES levels (AveSch-highSES) had the highest, lowest, and average mean levels of students’ science achievement and enjoyment in learning science respectively (Table 4). ANOVA and Tamhane *post hoc* tests showed that differences in mean levels of these student outcomes among the latent classes and all pairwise comparisons were significant at the 0.01 level.

**TABLE 4:** Students’ science achievement and enjoyment in learning science for different latent classes.

	PV1	PV2	PV3	PV4	PV5	PV6	PV7	PV8	PV9	PV10	Enjoy
	
	*M* (*SE*)										
Schools with the highest levels of resources and most qualified teachers but average SES levels (EquippedSch-AveSES)	498.96 (0.25)	499.07 (0.25)	498.95 (0.25)	498.81 (0.25)	499.00 (0.25)	499.04 (0.25)	498.92 (0.25)	498.80 (0.25)	498.69 (0.25)	498.98 (0.25)	2.65 (0.00)
Schools with lowest levels of resources, least qualified teachers, and lowest SES levels (NeedySch-LowSES)	479.96 (0.46)	479.62 (0.46)	479.62 (0.46)	479.62 (0.46)	479.72 (0.46)	479.91 (0.46)	479.89 (0.46)	470.60 (0.46)	479.39 (0.46)	479.87 (0.46)	2.57 (0.00)
Schools with average levels of resources and moderately qualified teachers but highest SES levels (AveSch-HighSES)	495.66 (0.44)	495.64 (0.44)	495.69 (0.44)	495.66 (0.44)	495.49 (0.44)	495.65 (0.44)	495.68 (0.44)	495.45 (0.44)	495.64 (0.44)	495.59 (0.44)	2.60 (0.00)

	**Comparison of mean levels**										

ANOVA (F statistics)	658.27**	687.51**	679.31**	670.85**	675.37**	664.88**	660.12**	670.33**	678.45**	664.51**	179.83**

	**Tamhane *post hoc* tests (Mean differences)**										

EquippedSch-AveSES vs. NeedySch-LowSES	19.00** (0.52)	19.45** (0.52)	19.32** (0.52)	19.19** (0.52)	19.28** (0.52)	19.13** (0.52)	19.03** (0.52)	19.20** (0.52)	19.29** (0.52)	19.11** (0.52)	0.08** (0.00)
EquippedSch-AveSES vs. AveSch-HighSES	3.30** (0.50)	3.43** (0.50)	3.26** (0.50)	3.15** (0.50)	3.51** (0.50)	3.39** (0.50)	3.24** (0.50)	3.35** (0.50)	3.04** (0.50)	3.39** (0.50)	0.05** (0.00)
NeedySch-LowSES vs. AveSch-HighSES	−15.71** (0.63)	−16.02** (0.63)	−16.07** (0.63)	−16.04** (0.63)	−15.77** (0.63)	−15.74** (0.63)	−15.79** (0.63)	−15.85** (0.63)	−16.25** (0.63)	−15.73** (0.63)	−0.03** (0.01)

*^∗∗^p < 0.01.*

### Relationships Between School Leadership and Students’ Science Learning

Table 5 summarizes HLM results for relationships between school leadership and the science achievement of students from schools with the highest levels of resources and most qualified teachers but average SES levels (EquippedSch-AveSES). Results showed that after controlling for students’ gender (Male) and SES (StuSES) and CHC, Professional-Devt (β = −7.70, *p* < 0.01) and Empowerment (β = −3.63, *p* < 0.05) were negatively associated with students’ science achievement. In contrast, Envisioning (β = −3.67, *p* = 0.07) and Instructional-Mgmt (β = −2.39, *p* = 0.10) were not related to students’ science achievement.

**TABLE 5:** School leadership and students’ science achievement for schools with highest levels of resources and most qualified teachers but average SES levels (EquippedSch-AveSES).

Parameter	Model 1	Model 2	Model 3A	Model 3B	Model 3C	Model 3D
**Fixed effects**						
Intercept	482.75**(6.25)	484.48**(5.16)	483.85**(5.14)	483.97**(5.08)	483.88**(4.96)	483.96**(5.13)
**Student variables**						
Male		6.00**(1.60)	5.99**(1.60)	6.00**(1.60)	6.00**(1.61)	6.00**(1.60)
StuSES		20.79**(1.31)	20.79**(1.31)	20.80**(1.30)	20.78**(1.30)	20.78**(1.31)
**School variables**						
Envisioning			−3.67 (2.05)			
Instructional-Mgmt				−2.39 (1.45)		
Professional-Devt					−7.70**(1.50)	
Empowerment						−3.63*(1.68)
**Country variable**						
CHC		39.76**(12.62)	38.33**(11.58)	38.24**(11.66)	37.99**(11.71)	39.65**(11.42)
**Intercepts**						
Level 1	6,396.98	6,151.70	6,151.66	6,151.61	6,151.43	6,151.58
Level 2	3,087.84**	2,364.00**	2,356.11**	2,359.34**	2,306.45**	2,353.45**
Level 3	1,285.47**	814.31**	789.20**	799.49**	750.87**	803.72**
**% variance**						
Level 1	59.39	65.93	66.17	66.07	66.80	66.08
Level 2	28.67	25.34	25.34	25.34	25.05	25.28
Level 3	11.94	8.73	8.49	8.59	8.15	8.63

*Standard errors in parentheses. ^∗^p < 0.05; ^∗∗^p < 0.01.*

Table 6 summarizes HLM results for relationships between school leadership and students’ enjoyment of science in schools with the highest levels of resources and most qualified teachers but average SES levels (EquippedSch-AveSES). After controlling for students’ gender (Male) and SES (StuSES) and CHC, none of the school leadership variables was significantly related to enjoyment at the 0.05 level (Envisioning, β = −0.00, *p* = 0.91; Instructional-Mgmt, β = −0.00, *p* = 0.58; Professional-Devt, β = −0.01, *p* = 0.13; Empowerment, β = −0.00, *p* = 0.76).

**TABLE 6:** School leadership and students’ enjoyment of science for schools with highest levels of resources and most qualified teachers but average SES levels (EquippedSch-AveSES).

Parameter	Model 4	Model 5	Model 6A	Model 6B	Model 6C	Model 6D
**Fixed effects**						
Intercept	2.61**(0.02)	2.62**(0.02)	2.62**(0.02)	2.62**(0.02)	2.62**(0.02)	2.62**(0.02)
**Student variables**						
Male		0.08**(0.01)	0.08**(0.01)	0.08**(0.01)	0.08**(0.01)	0.08**(0.01)
StuSES		0.10**(0.01)	0.10**(0.01)	0.10**(0.01)	0.10**(0.01)	0.10**(0.01)
**School variables**						
Envisioning			−0.00 (0.01)			
Instructional-Mgmt				−0.00 (0.00)		
Professional-Devt					−0.01 (0.00)	
Empowerment						−0.00 (0.01)
**Country variable**						
CHC		−0.19**(0.05)	−0.19**(0.05)	−0.19**(0.05)	−0.19**(0.05)	−0.19**(0.05)
**Intercepts**						
Level 1	0.60	0.59	0.59	0.59	0.59	0.59
Level 2	0.04**	0.03**	0.03**	0.03**	0.03**	0.03**
Level 3	0.02**	0.02**	0.02**	0.02**	0.02**	0.02**
**% variance**						
Level 1	90.91	92.19	92.19	92.19	92.19	92.19
Level 2	6.06	4.69	4.69	4.69	4.69	4.69
Level 3	3.03	3.13	3.13	3.13	3.13	3.13

*Standard errors in parentheses. ^∗∗^p < 0.01.*

Next, Table 7 summarizes HLM results for relationships between school leadership and the science achievement of students from schools with lowest levels of resources, least qualified teachers, and lowest SES levels (NeedySch-LowSES). Results showed that after controlling for students’ gender (Male) and SES (StuSES) and CHC, Professional-Devt (β = −6.75, *p* < 0.01) was negatively associated with students’ science achievement. The other three school leadership variables were not related to students’ science achievement (Envisioning, β = −3.27, *p* = 0.27; Instructional-Mgmt, β = −0.46, *p* = 0.81; Empowerment, β = −3.52, *p* = 0.13).

**TABLE 7:** School leadership and students’ science achievement for schools with lowest levels of resources, least qualified teachers, and lowest SES levels (NeedySch-LowSES).

Parameter	Model 1	Model 2	Model 3A	Model 3B	Model 3C	Model 3D
**Fixed effects**						
Intercept	464.49**(6.58)	465.68**(5.88)	465.49**(5.81)	465.61**(5.93)	465.82**(5.66)	465.44**(5.89)
**Student variables**						
Male		7.08**(1.94)	7.08**(1.93)	7.08**(1.93)	7.09**(1.94)	7.10**(1.93)
StuSES		15.29**(1.54)	15.29**(1.54)	15.29**(1.54)	15.27**(1.54)	15.28**(1.54)
**School variables**						
Envisioning			−3.27 (2.97)			
Instructional-Mgmt				−0.46 (1.91)		
Professional-Devt					−6.75**(2.01)	
Empowerment						−3.35 (2.22)
**Country variable**						
CHC		33.40*(12.15)	31.98**(11.56)	33.13*(12.04)	31.12**(10.78)	33.11**(11.29)
**Intercepts**						
Level 1	5,721.90	5,581.76	5,581.87	5,581.77	5,581.61	5,581.73
Level 2	3,167.71**	2,698.37**	2,692.61**	2,697.97**	2,659.10**	2,689.06**
Level 3	1,253.74**	915.22**	877.44**	912.23**	848.20**	894.34**
**% variance**						
Level 1	56.41	60.70	60.99	60.72	61.41	60.90
Level 2	31.23	29.34	29.42	29.35	29.26	29.34
Level 3	12.36	9.95	9.59	9.92	9.33	9.76

*Standard errors in parentheses. ^∗^p < 0.05; ^∗∗^p < 0.01.*

Table 8 summarizes HLM results for relationship between school leadership and students’ enjoyment of science in schools with lowest levels of resources, least qualified teachers, and lowest SES levels (NeedySch-LowSES). After controlling for students’ gender (Male) and SES (StuSES) and CHC, only Instructional-Mgmt was significantly related to enjoyment at the 0.05 level (β = 0.03, *p* < 0.05) whereas the other three school leadership variables were not (Envisioning, β = 0.02, *p* = 0.16; Professional-Devt, β = 0.00, *p* = 0.73; Empowerment, β = 0.00, *p* = 0.87).

**TABLE 8:** School leadership and students’ enjoyment of science for schools with lowest levels of resources, least qualified teachers, and lowest SES levels (NeedySch-LowSES).

Parameter	Model 4	Model 5	Model 6A	Model 6B	Model 6C	Model 6D
**Fixed effects**						
Intercept	2.54**(0.02)	2.55**(0.02)	2.55**(0.02)	2.56**(0.02)	2.55**(0.02)	2.55**(0.03)
**Student variables**						
Male		0.09**(0.02)	0.09**(0.02)	0.09**(0.02)	0.09**(0.02)	0.09**(0.02)
StuSES		0.08**(0.01)	0.08**(0.01)	0.09**(0.01)	0.08**(0.01)	0.08**(0.01)
**School variables**						
Envisioning			0.02 (0.02)			
Instructional-Mgmt				0.03*(0.01)		
Professional-Devt					0.00 (0.01)	
Empowerment						0.00(0.01)
**Country variable**						
CHC		−0.19**(0.05)	−0.18**(0.05)	−0.18**(0.05)	−0.19**(0.05)	−0.19**(0.05)
**Intercepts**						
Level 1	0.59	0.59	0.59	0.59	0.59	0.59
Level 2	0.04**	0.04**	0.04**	0.04**	0.04**	0.04**
Level 3	0.02**	0.02**	0.02**	0.02**	0.02**	0.02**
**% variance**						
Level 1	90.77	90.77	90.77	90.77	90.77	90.77
Level 2	6.15	6.15	6.15	6.15	6.15	6.15
Level 3	3.08	3.08	3.08	3.08	3.08	3.08

*Standard errors in parentheses. ^∗^p < 0.05; ^∗∗^p < 0.01.*

Moving on, Table 9 summarizes HLM results for relationships between school leadership and the science achievement of students from schools with average levels of resources and moderately qualified teachers but highest SES levels (AveSch-HighSES). Results showed that after controlling for students’ gender (Male) and SES (StuSES) and CHC, only Professional-Devt (β = −6.58, *p* < 0.01) was negatively associated with students’ science achievement whereas Envisioning (β = −4.17, *p* = 0.07), Instructional-Mgmt (β = −2.27, *p* = 0.16), and Empowerment (β = −2.62, *p* = 0.20) were not.

**TABLE 9:** School leadership and students’ science achievement for schools with average levels of resources and moderately qualified teachers but highest SES levels (AveSch-HighSES).

Parameter	Model 1	Model 2	Model 3A	Model 3B	Model 3C	Model 3D
**Fixed effects**						
Intercept	476.10**(6.52)	478.55**(5.52)	478.63**(5.38)	478.56**(5.45)	478.90**(5.56)	478.66**(5.43)
**Student variables**						
Male		6.83**(2.19)	6.83**(2.19)	6.83**(2.20)	6.85**(2.20)	6.84**(2.20)
StuSES		17.02**(2.07)	17.02**(2.07)	17.02**(2.07)	16.99**(2.07)	17.02**(2.07)
**School variables**						
Envisioning			−4.17 (2.30)			
Instructional-Mgmt				−2.27 (1.61)		
Professional-Devt					−6.58**(1.63)	
Empowerment						−2.62 (2.03)
**Country variable**						
CHC		40.02**(8.47)	37.11**(7.31)	37.61**(7.42)	36.12**(8.87)	39.29**(7.86)
**Intercepts**						
Level 1	6,138.74	5,982.62	5,982.49	5,982.61	5,982.53	5,982.47
Level 2	3,118.62**	2,601.10**	2,594.13**	2,596.92**	2,559.92**	2,596.78**
Level 3	1,151.92**	781.38**	750.59**	770.09**	768.80**	775.30**
**% variance**						
Level 1	58.97	63.88	64.14	63.99	64.25	63.95
Level 2	29.96	27.77	27.81	27.78	27.49	27.76
Level 3	11.07	8.34	8.05	8.24	8.26	8.29

*Standard errors in parentheses. ^∗∗^p < 0.01.*

Lastly, Table 10 summarizes HLM results for relationships between school leadership and students’ enjoyment of science in schools with average levels of resources and moderately qualified teachers but highest SES levels (AveSch-HighSES). After controlling for students’ gender (Male) and SES (StuSES) and CHC, only Instructional-Mgmt (β = 0.02, *p* < 0.05) was significantly related to students’ enjoyment. In contrast, the other three school leadership variables (Envisioning, β = 0.01, *p* = 0.19; Professional-Devt, β = −0.00, *p* = 0.60; Empowerment, β = 0.01, *p* = 0.40) were not associated with students’ enjoyment.

**TABLE 10:** School leadership and students’ enjoyment of science for schools with average levels of resources and moderately qualified teachers but highest SES levels (AveSch-HighSES).

Parameter	Model 4	Model 5	Model 6A	Model 6B	Model 6C	Model 6D
**Fixed effects**						
Intercept	2.60**(0.03)	2.61**(0.02)	2.61**(0.02)	2.61**(0.02)	2.61**(0.02)	2.61**(0.02)
**Student variables**						
Male		0.10**(0.03)	0.10**(0.03)	0.10**(0.03)	0.10**(0.03)	0.10**(0.03)
StuSES		0.09**(0.01)	0.09**(0.01)	0.09**(0.01)	0.09**(0.01)	0.09**(0.01)
**School variables**						
Envisioning			0.01 (0.01)			
Instructional-Mgmt				0.02*(0.01)		
Professional-Devt					−0.00 (0.01)	
Empowerment						0.01 (0.01)
**Country variable**						
CHC		−0.15**(0.05)	−0.14**(0.04)	−0.13**(0.04)	−0.15**(0.05)	−0.15**(0.05)
**Intercepts**						
Level 1	0.60	0.59	0.59	0.59	0.59	0.59
Level 2	0.04**	0.04**	0.04**	0.04**	0.04**	0.04**
Level 3	0.02**	0.02**	0.02**	0.02**	0.02**	0.02**
**% variance**						
Level 1	90.91	90.77	90.77	90.77	90.77	90.77
Level 2	6.06	6.15	6.15	6.15	6.15	6.15
Level 3	3.03	3.08	3.08	3.08	3.08	3.08

*Standard errors in parentheses. ^∗^p < 0.05; ^∗∗^p < 0.01.*

## Discussion

### School SES and Different Types of School Resources

Results from the present study showed that there were three types of schools differing in their contexts as measured by the adequacy of science resources, proportion of qualified science teachers, and school SES. Interestingly, schools with the highest SES were not those with the highest level of qualified science teachers and science resources. This finding may arise because schools vary in the specific types of resources that they have, and high-SES parents may send their children to attend schools with the resources that they value. For example, in Japan, some royal families and very renowned political leaders may aspire their children to study in Gakushûin in order to be socially connected to the most powerful elite in the Japanese society. These schools are endowed in its socio-political capital and general resources but not necessarily science resources. In the same vein, parents in the UK who are alumni of Eton College may enroll their children in their alma mater in view of the prospect for economic connections instead of other considerations such as the adequacy of science resources. Therefore, the present study does not categorically “refute” the association between school SES and resources reported in some studies (Willms, 2010; Liu et al., 2015) but instead point to possible nuances in the specific types of resources that schools have. Future studies can ascertain the different types of resources that characterize high-SES schools.

### Inextricable Relationships Between School Contexts and Leadership

Results from the present study also showed that levels of the four principal leadership variables varied with the three types of schools. These results are consistent with the refrain in the school leadership scholarship regarding the need to examine leadership effects in the school context that the leadership is enacted (Hallinger, 2018). However, we do not have a clear understanding of how school contexts and leadership are related to each other. Contexts may shape leadership (as is assumed in this study), so school leaders have to adapt their practice to the school environment that they are operating in Wasserman et al. (2010). Alternatively, leadership may shape contexts, so school leaders have the agency to develop their “ideal” school environment to support their school improvement plans (Hendriks and Scheerens, 2013). Lastly, it can be the case that contexts and leadership may exert a mutual influence on each other. Obviously, the three scenarios carry different implications for school leadership, so future research can clarify the causal relationship between school contexts and leadership.

#### Envisioning, Instructional Management, and Empowerment in More-Endowed School Contexts

In terms of specificity, levels of principals’ envisioning, instructional management, and empowerment were the highest in well-endowed schools (i.e., highest proportion of qualified science teachers and adequate science resources) as compared to the other two types of schools. Principals of schools staffed by qualified teachers may be more likely to focus on setting shared goals (i.e., envisioning) because these teachers have greater capacity to achieve these goals (Notman and Henry, 2011). Principals of well-resourced schools may also be more involved in envisioning because there are adequate resources for realizing school academic goals. The importance of school resources is highlighted by Murphy and Torre (2015) who argued for the alignment between school visions, improvement, and organization including budgets (for resource allocation), operating procedures, structures, and policies.

Next, principals leading schools with more qualified teachers are more likely to focus on instructional management since these teachers are equipped to implement instructional initiatives that promote student-centered pedagogies. Principals leading well-resourced schools may also be more likely to focus on instructional management given the availability of resources to support the implementation of innovative pedagogies (Cohen et al., 2003). The importance of school resources can be inferred from Chang et al. (2008) study of Taiwanese elementary schools which reported that the successful implementation of school plans for technology-enabled instruction required adequate budgets, technological, and other resources. In the case of science education, updated science teaching resources are especially crucial for teachers to deliver effective student-centered lessons.

Principals leading schools with qualified teachers may be more likely to empower teachers to leverage the professional knowledge of these teachers to improve the school. This relationship is evident in Lochmiller and Acker-Hocevar’s (2016) qualitative study of US public high school administrators which found that principals when confronted with a lack of content knowledge in specialized subject areas such as mathematics and science resorted to hiring teachers who could teach effectively and work collaboratively, allocating resources to support teacher collaboration, and providing professional development.

#### Importance of Promoting Teachers’ Professional Development Across Different School Contexts

The finding for the leadership practice of principals promoting teachers’ professional development was more nuanced − principals were more involved in promoting teachers’ professional development in well-endowed schools (i.e., schools with qualified teachers and adequate resources) than in schools with average levels of these resources but there was no difference between the most and least endowed schools. Compared to the pattern of results for the other leadership practices (envisioning, instructional management, empowerment) in different types of school contexts, these results suggest that school leaders are more likely to focus on teachers’ professional development regardless of their levels of school resources and teacher quality. This leadership imperative reflects the difficulty of hiring new teachers as compared to training existing ones (Hitt and Tucker, 2016) and hence the need for principals to make the best use of available teacher capacity in the school. For example, Lai’s (2014) qualitative research in Hong Kong found that principals promoted teachers’ professional development by sending teachers to attend external courses when there were teacher resource and institutional constraints. Professional development is particularly helpful if teachers have specific developmental needs to be addressed. For example, principals can also help struggling teachers by providing professional development programs, guidance in classroom management, and organizational, financial, and human support (Yariv and Kass, 2019). Indeed, teachers who undergo professional development get to become more cohesive, professional, competent, and efficacious (Hendriks and Scheerens, 2013). These teachers can contribute to the school academic and improvement capacity.

### Principal Leadership and Students’ Holistic Learning Outcomes

The present study examined academic and non-academic student learning outcomes in science, namely science achievement and enjoyment in learning science. This more comprehensive conception of student learning, beyond academic achievement alone, is in line with the aims of high-performing education systems worldwide to equip students with knowledge, competencies, and skills that are fit for purpose in the 21st century knowledge-based economies. Notwithstanding the salience of holistic learning outcomes, there are few studies examining the contribution of principal leadership to different students’ learning attitudes. Some leadership researchers only focused on specific students’ variables [e.g., self-efficacy in Zheng et al. (2017); student engagement in Leithwood and Jantzi (2000); self-concepts, participation, and engagement in Silins and Mulford (2002)]. For example, Zheng et al. (2017) reported that, compared to other leadership factors pertaining to visibility and direct participation, organization of school environment, planning and personnel, and external relations, principals’ role in developing teaching-learning most highly predicted grade 8 students’ reading achievement and self-efficacy in China. However, Kruger et al. (2007) failed to find a relationship between principal leadership and student commitment as measured by students’ perceptions of their relationships with teachers, of the school organization, and of the school culture. The present study therefore addresses the lacuna in our knowledge base on whether principal leadership practices can improve students’ learning attitudes and achievement in the area of science. Additionally, results from the present study showing that only instructional management exercised in schools with lowest levels of resources, least qualified teachers, and lowest SES levels (NeedySch-LowSES) and schools with average levels of resources and moderately qualified teachers but highest SES levels (AveSch-HighSES) provide nuanced insights on the types of specific distal antecedents (principal leadership and school contexts) that may influence students’ control and value appraisals and consequently their enjoyment of science learning. These contextual insights complement the set of psychological variables in the control-value theory that researchers are increasingly using to explain student experiences of emotions in their learning (Pekrun et al., 2007; Mercan, 2020).

### Instructional Management for Promoting Educational Equity

The present study showed that among the four leadership practices, only instructional management was positively related to students’ enjoyment of science learning in schools with lowest levels of resources, least qualified teachers, and lowest SES levels (NeedySch-LowSES) and schools with average levels of resources and moderately qualified teachers but highest SES levels (AveSch-HighSES) but not in schools with highest levels of resources and most qualified teachers but average levels of SES (EquippedSch-AveSES); none of the leadership practices was significantly related to students’ science achievement. Instructional management can contribute to students’ enjoyment of science learning when teachers’ instructional practices are informed by the latest research focusing on student-centered learning, when principals emphasize the importance of teachers developing students’ critical and creative thinking capacities (beyond textbook knowledge), and when principals recognize teachers’ efforts to provide effective student-centered pedagogies in their teaching. These aspects of instructional management are encapsulated in the leadership items used to measure principals’ instructional management practices in the present study.

The finding that principals’ instructional management was only positively related to students’ science enjoyment in less-endowed (schools with lowest levels of resources, least qualified teachers, and lowest SES levels (NeedySch-LowSES) and schools with average levels of resources and moderately qualified teachers but highest SES levels (AveSch-HighSES) is consistent with that reported in an evolving body of literature. For example, Tan’s (2018a) analysis of PISA 2012 data found that principal instructional leadership was most strongly associated with the mathematics achievement of students who attended the least-resourced schools in OECD countries; these students were also from the lowest SES families, had the lowest prior achievement level, had parents with the lowest academic expectations of schools.

There are many reasons why principals’ instructional management may contribute more to student learning in less-endowed schools. First, principals in these schools may exercise greater instructional control and are more focused on teaching-and-learning than building relationships (Hallinger and Murphy, 1986). Some studies indicate that principals also leveraged on collaborative instructional leadership focusing on teaching-and-learning (Hallinger and Heck, 2011). Second, principals may facilitate more instructional discussions among teachers, protect teachers from classroom disruptions, leverage test results more frequently to improve instructional programs, establish more systematic monitoring of student progress, and communicate instructional goals to teachers more effectively (Heck, 1992). Lastly, from a school improvement perspective, Day et al.’ (2016) mixed-method, longitudinal study of effective and improving English schools underscored the need for principals to have high levels of expectations in classroom teaching, emphasize student behavior and achievement, conduct more classroom observations, and coach less effective teachers.

The finding that principals’ instructional management was positively related to student learning in less-endowed schools contributes to the policy discourse on school equity. There is an expectation that effective school systems achieve high levels of student performance (educational excellence) for different groups of students (educational equity) (Schleicher, 2009). In the context of the present study, greater equity means closing the learning gap between students in advantaged and disadvantaged school contexts. Therefore, principals in less-endowed schools can focus more on instructional management (vis-à-vis other leadership practices) to improve student learning even in less-endowed school contexts (Dimmock and Tan, 2016).

### Negative Relationships Between Enhancing Teacher Capacity and Student Learning

The present study examines two principal leadership practices that are aimed at enhancing teacher capacity, namely promoting professional development and teacher empowerment. Results showed that the two leadership practices were not positively related to student learning. Instead, results from the present study showed that in all three school contexts, students whose principals promoted professional development for teachers had lower levels of science achievement.

These results may arise because principals have limited time and energies to manage myriad school needs (Goldring et al., 2008; May et al., 2012), so if they focus on teachers’ professional development they will have less capacity to spearhead instructional initiatives which may have a more direct impact on student learning. More fundamentally, it is important to ascertain what drives higher levels of teachers’ professional development in the first place. If schools suffer from a deficit in teacher quality and principals attempt to address this capacity issue through professional development, then it takes time for effects of enhanced teacher capacity via professional development to manifest in student learning. Indeed, teachers need to change their pedagogical beliefs and practices simultaneously to effect changes in students’ learning outcomes (Clarke and Hollingsworth, 2002). Therefore, if teacher capacity constraints are severe and professional development fails to change complex systems of influences simultaneously, then student learning may not improve (Opfer and Pedder, 2011). Another possible reason to explain the negative relationships between principals’ promotion of teachers’ professional development and student learning is that some teachers may perceive greater professional development as undue influences to shape their professional practice. This argument reinforces Opfer and Pedder’s (2011) thesis to appreciate the complex interplay among school factors, teacher factors, and the learning activity that collectively impact the effectiveness of teachers’ professional development. If this is so, teachers who are asked to attend professional development may perceive an erosion of professional autonomy and be less motivated (Hallinger and Lu, 2014). The decreased motivation may impact the quality of teaching adversely.

As for teacher empowerment, results showed that this leadership practice, just as in the case for teachers’ professional development, was also not positively related to student achievement. Specifically, teacher empowerment was negatively related to student achievement in EquippedSch-AveSES schools and not related to student achievement in the other two types of schools. These results may happen because teachers who are expected to contribute to organizational improvement may not be able to commit their energies and resources to improving teaching-and-learning. The tension between leadership and teaching responsibilities is evident in Brooks et al. (2004) study where teacher leaders perceived their leadership responsibilities as “a source of frustration that pried them from the essential, instructional tasks of their profession” (p. 253). As a result, students may not benefit directly from increased teacher empowerment.

### Contributions, Limitations, and Future Research

The present study elucidates the different types of contexts that schools operate in and clarifies how some leadership practices differentially impact students’ science learning depending on these school contexts. Data from 248,620 students and 9,370 school principals in 35 OECD countries who participated in PISA 2015 were analyzed using LPA, ANOVA and Tamhane post-hoc comparisons, and three-level HLM. The study contributes to theory and practice in three ways.

First, it is one of the few studies to provide empirical evidence that schools do not operate in homogeneous contexts by clarifying how these different school contexts look like in terms of the availability of science resources, quality of science teachers, and school SES. Among the three types of school contexts identified in the LPA, schools with lowest levels of resources, least qualified teachers, and lowest SES levels (NeedySch-LowSES) represents the most challenging contexts that 19.02% of the schools in the sample operate in. These schools are confronted with having less science resources, less qualified science teachers, and students from lower-SES families who are likely to receive less parental support for their learning. The study makes a second contribution by identifying leadership practices associated with specific types of school contexts. For example, results showed principals were more likely to have envisioning, instructional management, and teacher empowerment in schools that had the most science resources and best science teacher quality (EquippedSch-AveSES). Future studies can unravel what principals focus on in their leadership in less-endowed schools and whether these leadership priorities contribute to student learning. The study makes a third contribution by identifying principals’ instructional management as being more effective than promoting professional development or empowering teachers for students’ science learning in schools with less science resources and lower science teacher quality [schools with lowest levels of resources, least qualified teachers, and lowest SES levels (NeedySch-LowSES), schools with average levels of resources and moderately qualified teachers but highest SES levels (AveSch-HighSES)]. Instructional management thus seems to have a compensatory effect on students’ learning in less-endowed schools. However, professional development and empowerment are means to addressing teachers’ competence and autonomy needs (Eyal and Roth, 2011; Shepherd-Jones and Salisbury-Glennon, 2018) and thereby, building teacher capacity which will in the long term also benefit student learning. How then should principals strike a balance between focusing on instructional management and building teacher capacity? Future research can examine how principals negotiate these different leadership priorities.

As with all empirical studies, results from the present study should be read with some limitations in mind. First, the PISA sample comprised only 15-year-old students who were mostly in Grade 10 (55.9%) with the rest were from Grades 7−13, so results reported are applicable only to this student population. Second, it examined only four core leadership practices in Leithwood et al. (2006) conceptualization, so future studies can examine other leadership practices. Third, the study relied on principals’ self-reported data for their leadership practices, so future studies can complement these with teacher-reported data to reduce bias (Urick, 2016). Fourth, the focus on students’ learning in science instead of other subject areas assumes that schools generally value science learning but there are schools which may value learning in other domains (e.g., aesthetics in Waldorf School) as much as, if not more than, in science. Therefore, results from the present study have to be interpreted with this caveat in mind. Lastly, the data analyzed were correlational in nature, so causal inferences should be made with caution. Causal, or at least longitudinal, research designs in future research can be used to ascertain the relationships reported.

## Data Availability Statement

Publicly available datasets were analyzed in this study. This data can be found here: https://www.oecd.org/pisa/data/2015database/.

## Ethics Statement

Ethical review and approval was not required for the study on human participants in accordance with the local legislation and institutional requirements. Written informed consent to participate in this study was provided by the participants’ legal guardian/next of kin.

## Author Contributions

CT conceptualized the study and wrote 60% of the article (including Sections “Introduction, Materials and Methods, Results, and Conclusion”). PL contributed to the literature review. WW contributed to the discussion of the article. All authors contributed to the article and approved the submitted version.

## Conflict of Interest

The authors declare that the research was conducted in the absence of any commercial or financial relationships that could be construed as a potential conflict of interest.

## Footnotes

1 https://read.oecd-ilibrary.org/education/pisa-2015-assessment-and-analytical-framework/pisa-2015-background-questionnaires_9789264281820-9-en#page37

2 https://read.oecd-ilibrary.org/education/pisa-2015-assessment-and-analytical-framework/pisa-2015-science-framework_9789264281820-3-en#page1
