# Supplementary material for: Different Patterns of Relationships Between Principal Leadership and 15-Year-Old Students’ Science Learning: How School Resources, Teacher Quality, and School Socioeconomic Status Make a Difference
Source: Front Psychol. 2020 Aug 27;11:2257. doi: 10.3389/fpsyg.2020.02257 (PMC7481831; doi:10.3389/fpsyg.2020.02257)
Supplement: Supplementary file 1 [file Table_1.DOCX]

School principals are confronted with myriad responsibilities in their leadership. These responsibilities include improving instructional quality and building teacher capacity to improve student learning. In addition, they have to adapt their leadership to their unique school contexts. However, there is a paucity of research that compares the relative effectiveness of leadership practices in different school contexts. Therefore, we do not know what types of leadership practices are effective in specific school contexts. The present study addresses this gap by examining data on school contexts, principal practices, and students’ science learning from a large sample of almost 250,000 fifteen-year-old students and more than 9,000 school principals from 35 developed countries who participated in PISA 2015. Results showed that there were three types of contexts that schools operated in and that there were differences in leadership effectiveness in different school contexts. Importantly, student learning in schools with less resources and poorer teacher quality benefited from principals who focused on instructional management, thereby indicating the potential of this principal function to contribute to educational equity. In contrast, student learning in all three school contexts did not benefit from principals focusing on envisioning, teachers’ professional development, or empowerment.
